# Dielectric characterization of paraelectric particle-loaded polymer matrix composites and commercial photoresins at W-band frequencies

**DOI:** 10.1016/j.heliyon.2023.e13458

**Published:** 2023-02-03

**Authors:** Michael Forstmeier, Mengxue Yuan, Steve Perini, Michael Lanagan, Brian Foley

**Affiliations:** a Department of Mechanical Engineering, The Pennsylvania State University, University Park 16802, USA; b Department of Engineering Science & Mechanics, The Pennsylvania State University, University Park 16802, USA; c Materials Research Institute, The Pennsylvania State University, University Park 16802, USA

**Keywords:** Polymer Matrix Composite (PMC), Designer-dielectric, Stereolithography, Radio frequency, Additive manufacturing

## Abstract

This work presents W-band (75–110 GHz) dielectric characterization of commercially available photoresins in their neat state, as well as in polymer matrix composite (PMC) mixtures with various loading concentrations of the paraelectric barium strontium titanate (BST). Due to difficulties 3D printing the BST-loaded PMC resins detailed within, a custom curing and casting process was used to fabricate testable PMC samples, which were synthesized to demonstrate the dielectric functionalization of the underlying polymer matrix. Dielectric characterization of the PMCs confirmed the functionalization of our composites when compared to the commercial photoresins. For example, a volumetric loading concentration of 25 vol % BST increased the dielectric permittivity (*ε_r_*) from 2.78 to 9.60 and the loss tangent (tanδ) from 0.022 to 0.114. These results indicate that the realization of UV-cured photoresins with “designer-dielectric” functionalization based on vol % of filler are strong candidates for use in stereolithography (SLA) 3D printing applications. To accomplish this, and with a special interest for radio/microwave/terahertz (RF/MW/THz) applications, we highlight the need for both (a) better photoresin matrix materials with lower intrinsic tanδ and (b) selection criteria related to the size/geometry and electronic properties of potential filler materials to maintain the printability of PMC photoresins in SLA systems.

## 1 Introduction

Additive Manufacturing (AM), often referred to as ‘3D printing’, is of great interest to the radio/microwave/terahertz (RF/MW/THz) design communities. AM has been implemented in relevant sub-foci ranging from planar transmission lines and antenna structures [1] to free-space, quasi-optical components [2]. These application spaces have begun to explore the use of AM because of its unique ability to rapidly prototype and fabricate complex geometries and features not possible with conventional production methods. Given current interest in 5th-generation (5G) communications systems and components, as well as the excitement related to future 6th-generation (6G) networks, the continued maturation of AM processes for RF/MW/THz applications has great potential to impact our increasingly interconnected society.

High-frequency component design and performance is largely determined by two design considerations, namely the accuracy of geometric features and the values of the electronic properties of the underlying materials. Because the design and ultimately the performance of a given component are strongly coupled to the accuracy of these geometric and material characteristics, it is critical that any proposed ‘Additive-for-RF’ approach can produce structures that meet the stringent expectations of this application space. More specifically, high-frequency structures must have adequate surface finishes [3,4], optimal dielectric constants [5,6], low losses [7], and precise geometries [8], regardless of the manufacturing method.

Certain AM techniques such as fused deposition modeling (FDM) and PolyJet printing have been used to fabricate components intended for X-band (8–12 GHz) operation, meeting several of the criteria mentioned previously [1]. However, if the intended operational frequency of the application space increases into the range of high-band 5G (26–40 GHz) or into the sub-THz range for 6G (95–300 GHz), the geometric features and required dimensional tolerances of a given component will shrink to the 10–100 μm level. AM processes, such as FDM, often do not possess the fabrication resolution necessary to satisfy the geometric requirements of high-frequency components.

Due to its excellent print resolution and accuracy, stereolithography (SLA) is an ideal AM method for RF/MW/THz applications. SLA is an AM technology wherein a focused, steerable UV light source is used to selectively cure the monomer chains of a liquid resin layer-by-layer into a hardened plastic part. Due to excellent control of the light source, SLA can obtain both layer and lateral resolutions on the order of ∼10s of μm. The accuracy of this fabrication method can be leveraged to address the geometric concerns of the RF/MW/THz communities.

Along with part geometry, material selection based on the electronic/dielectric properties of the underlying material is another critical aspect of the design of high-frequency components. In particular, the complex dielectric properties of the underlying material, namely the relative permittivity (*ε_r_*) and the loss tangent (tanδ), often dictate many of the geometric aspects of a high-frequency component. In other words, the complex dielectric properties of the underlying material influence the design and size of geometric features and therefore facilitate or hinder certain component functionalities (e.g., wave propagation, reflection, and attenuation, among others). As application frequencies approach 100 GHz and above, finding reliable data for *ε_r_* and tanδ of any standard material, not just those used in AM processes, can be quite a challenge. Recognizing the importance of this material data, there is a need for accurate, broadband dielectric characterization.

In situations where a particular geometric attribute may not be practical and/or manufacturable using a standard material, it would be highly desirable to use a ‘designer-dielectric’, a material class in which *ε_r_* and tanδ can be tailored to the ideal value(s) required by the application. One class of functionalized dielectric materials are the polymer matrix composites (PMCs), which are typically composed of a polymer base/matrix material and a nano/micro-particle filler [9]. Both ceramic and non-ceramic fillers have been used extensively to alter the thermal [10], mechanical [11], magnetic [3] and dielectric characteristics [[12], [13], [14], [15], [16]] of various polymer matrices for use in AM and/or other advanced manufacturing methods. While some studies have motivated the use of PMCs in 3D printing [9], a limited number have specifically motivated their use in SLA [17]. Furthermore, even fewer studies have investigated the dielectric properties of additively manufactured PMCs in the W-band (75–110 GHz) [13].

Given the immense opportunity for disruptive innovation in the RF/MW/THz range of the electromagnetic spectrum, this work explores the dielectric functionalization of PMCs composed of commercially available methacrylate-based photopolymer resins and a barium strontium titanate (Ba0.67Sr0.33TiO3, BST) paraelectric filler. Additional outcomes of this work include W-band dielectric characterization of several commercial photoresins produced by Formlabs, which are designed for and used in their SLA 3D printers. The data gathered in this work indicate that the SLA polymers tested are too lossy for most high frequency applications. Therefore, low loss resins must be designed if the high precision capabilities of SLA are to be leveraged by the RF/MW/THz design communities. Finally, stemming from the challenges of 3D printing BST-loaded PMCs as chronicled herein, recommendations are made to enhance the SLA-printability of novel PMCs.

## 2 Material & methods

### 2.1 Materials

The PMCs used in this work were two-part composite mixtures consisting of a polymer base/matrix and a ceramic filler. The matrix material for all PMCs studied here was a methacrylate-based, UV-curing photopolymer resin from Formlabs (Sommerville, MA) called Clear (V4). While Clear was the only resin used in the PMCs, several additional resins from Formlabs were also characterized at W-band frequencies to determine any resin-specific variations in the complex dielectric properties of Formlabs products. These selected resins included Black, Gray, White, Draft (V2), Model (V3), High Temperature (V2) and Ceramic (both in the pre/Green and post-sintered states).

The ceramic filler for the PMCs was paraelectric barium strontium titanate (Ba0.67Sr0.33TiO3, BST) from TPL Inc. (Albuquerque, NM). The BST particles that composed the TPL powder were spherically shaped and approximately 200 nm in diameter.

### 2.2 Commercial photoresin test samples

Samples fabricated from the as-received photoresins were printed with a Form2 SLA 3D printer from Formlabs. Three (3) structures made from each photoresin were printed and post-washed/cured according to the Formlabs resin guidelines. The ceramic structures, which were treated according to the Formlabs post-print ceramic sintering process, received a unique post-wash/cure cycle. The design dimensions of the samples were 40 mm × 40 mm x 2 mm (sample side lengths and thickness, respectively), and additional support structures were included to facilitate successful prints. These support structures were then removed following the post-cure process, yielding the final form of the square 3D printed samples.

### 2.3 Ceramic-infilled photocurable resin preparation

Ceramic-infilled photocurable resins of 5, 10, 20 and 25 vol % BST, each 200 mL in total volume, were prepared using a three-step loading and mixing procedure:

(1) Appropriate amounts of BST powder and Clear resin were combined to synthesize the concentrations studied in this work.

Following the combination of materials, a two-step mixing routine was implemented using a Thinky Mixer ARE-500. This mixing routine was performed under vacuum to eliminate unwanted bubbles in the mixture, a technique commonly employed in the preparation of ceramic-infilled photocurable resins [18]. Both the pre-mixing and final mixing phases had two spin cycles, which together make up the final two steps of the loading and mixing procedure:

(2) Pre-mixing (under vacuum): 500 rpm for 30 s, followed by 1000 rpm for 30 s.
(3) Final mixing (under vacuum): 500 rpm for 30 s, followed by 1000 rpm for 90 s.

After mixing, the ceramic-infilled photocurable resins were transferred into opaque containers to prevent any unintentional cross-linking due to ambient light exposure.

### 2.4 PMC solid sample fabrication

**Fig. 1:**
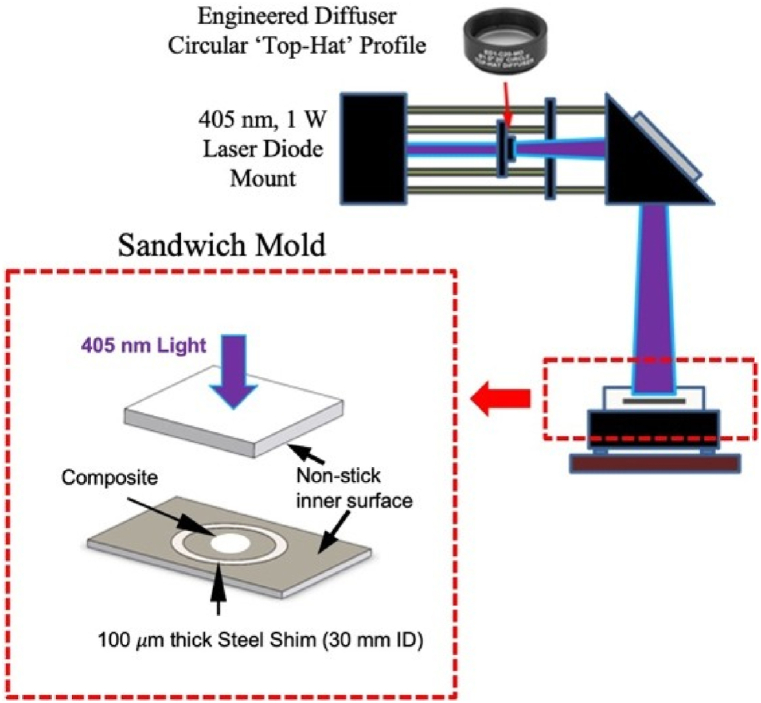
A schematic diagram of the laser curing apparatus and the casting mold used in the fabrication of BST-loaded samples.

Due to difficulties encountered with 3D printing BST-loaded PMCs directly, solid test samples were synthesized via a casting procedure similar to others found in the literature [18]. Ceramic-infilled photocurable resins were deposited into a circular casting mold, which was a 100 μm thick, 30 mm inner diameter Spring Steel shim (McMaster-Carr). The shim rested on top of a clear, flexible polymer sheet that was adhered to a glass slide. To promote uniform spreading, the mixtures were deposited near the center of the shim and sandwiched by a second piece of glass, which was also covered by a polymer sheet. In this manner, thin circular samples were formed for subsequent curing. A schematic diagram of the sandwich mold is shown in Fig. 1 (lower left).

After preparing the molds, the deposited ceramic-infilled photocurable resins were UV-cured in a custom curing apparatus. The sandwiched molds were uniformly illuminated by a 405 nm UV light, which was emitted by a 1 W diode laser source (Thorlabs). The uniform intensity profile in the illumination area was accomplished by using an Engineered Diffuser optic (Thorlabs) to transform the Gaussian intensity profile of the laser into the resultant ‘top-hat’ profile. Fig. 1 demonstrates a simplified schematic of the laser manipulation and curing process. Each slurry specimen was laser cured for as much as 12 min on both sides, ensuring complete curing of the PMC even at the highest BST loading concentrations. Each solidified specimen was then removed from the shim and rinsed in an IPA bath to remove any uncured resin. Three (3) solid samples were fabricated via this method for each vol % BST PMC mixture. When dry, the thickness of every cast sample was measured using a digital micrometer. In total, 15 thickness measurements were made on each sample to quantify mean thicknesses and standard deviations (s_d_).

### 2.5 Dielectric measurement procedure

**Fig. 2:**
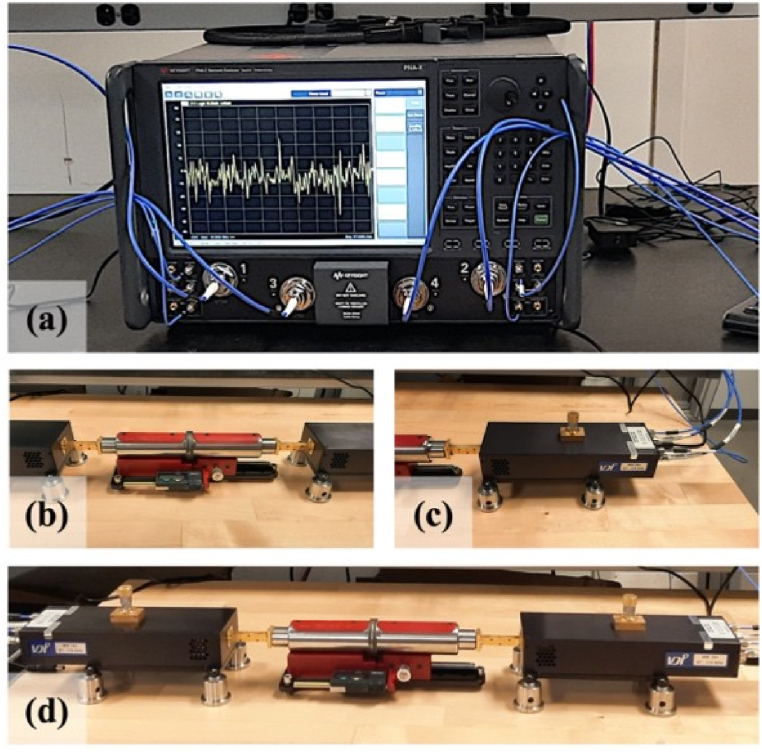
Laboratory images of the W-band dielectric characterization equipment: (a) PNA-x, (b) SWISSto12, (c) Extension Module, and (d) complete W-band testing assembly.

To perform dielectric characterization in the W-band, a Keysight Technologies PNA-X (N5742B) vector network analyzer (VNA) was used in conjunction with two Virginia Diodes, Inc. (VDI) W-band frequency extension modules and a material characterization kit (MCK) from SWISSto12. Fig. 2(a–d) displays these components.

Because the Keysight Technologies PNA-X is limited to a frequency range of 10 MHz–67 GHz, the VDI modules were used to extend its operating frequency into the W-band. The extension modules were connected to the MCK W-band test fixture, which enabled accurate measurement of the complex dielectric properties of the samples studied in this work. The test fixture is essentially composed of two corrugated horn antennas (output aperture ∼18 mm) that sandwich the test sample and guide the EM waves into/through the material under test. By characterizing the scattering parameters (S-parameters) associated with frequency-dependent transmission (S21) and reflection (S11) of EM waves interacting with the sample, the relative permittivity (*ε_r_*) and the loss tangent (tanδ) of a given sample can be determined.

An important part of any transmission/reflection-based dielectric characterization experiment is the measurement equipment calibration method. The MCK test fixture employs a gated-reflect-line standard to calibrate the overall system. A thorough description of the standard is found in the literature [19]; in brief, the system utilizes the time-domain analysis capabilities of the VNA to ‘gate’ the primary transmission/reflection signals, thereby rejecting erroneous information carried in multi-path reflections in the measurement system. Through the use of an appropriate gate width (here 400 ps), a simple normalization of the S11 (using a perfect reflector, realized with a polished aluminum sample, 1 mm thick) and S21 (no sample, horns firmly mated together at the reference plane) magnitudes and phase responses is adequate to calibrate the measurement system.

Following calibration, both data acquisition and subsequent dielectric analyses were conducted via the SWISSto12 control software included with the MCK. The MCK software only requires the user to provide the thickness of the sample under test in order to calculate *ε_r_* and tanδ for the material. In the case of this study, the average thickness of each test sample, as determined by post-fabrication micrometer measurements, was used in the analysis. In this work, the values of *ε_r_* and tanδ are assumed to remain relatively constant over the W-band range, with no resonances or sharp spectral features observed during testing. Therefore, the W-band loss characteristics presented in this work were computed in the SWISSto12 control software as frequency band averages. Multiple measurements were made on each solid sample (3D printed test coupons and cast PMC samples alike) to ameliorate any uncertainty associated with measurement repeatability in the analysis. The uncertainty associated with sample variability was addressed by synthesizing several samples of each material type.

It was determined that the uncertainty in the thickness of a given sample is by far the dominant factor behind the observed uncertainties in *ε_r_* and tanδ from the MCK measurements. For each scan taken with the MCK, the sample thickness input to the software was varied by ±2 s_d_ to capture the impact of thickness variation on the fitted dielectric parameters. The error bars shown in subsequent plots represent a combination of the various sources of uncertainty via a root-sum-squares (RSS) approach. The considerable error bars at higher loading concentrations are likely due in part to poor dispersion and settling of heavy ceramic particles in the resin mixture. This limitation can be ameliorated by introducing additives to the resin mixtures that are designed to help with particle dispersion.

## 3 Results and discussion

### 3.1 The dielectric analysis of neat polymers

**Fig. 3:**
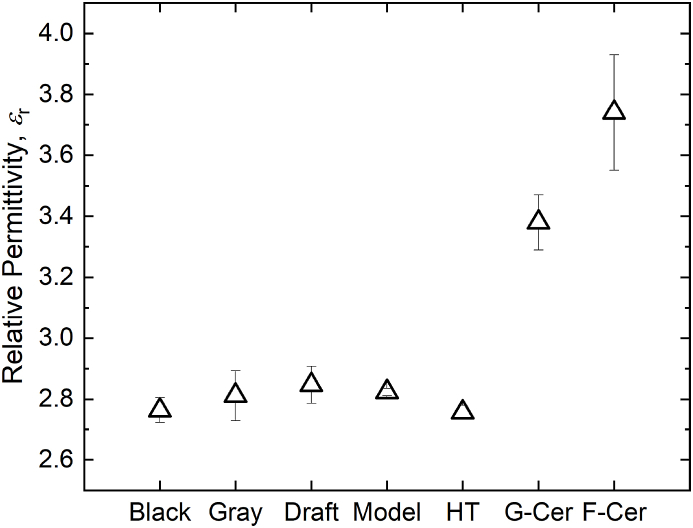
The W-band dielectric permittivity of several commercially available Formlabs SLA resins.

Prior to assessing the dielectric functionalization of BST-loaded PMC resins, it was important to first characterize the dielectric properties of the underlying commercial photoresin matrices. To this end, Formlabs photoresins were characterized in their neat state, i.e. a condition in which the polymer matrix remained unloaded by BST particles. This condition is hereafter referred to as ‘polymer-only’. Although Clear resin was the only Formlabs resin used for the fabrication and testing of the BST PMC samples, several additional photoresins from Formlabs were printed and characterized in the W-band. The resin-specific dielectric data gathered from these experiments will be of use to those designing RF/MW/THz elements. Further, this data set will enable RF/MW/THz designers to select from a wider array of SLA materials for the fabrication of their high-frequency components. Table 1 contains the measured *ε_r_* and tanδ values in the W-band for each of the eight photoresins considered, including measurements for the ceramic resin in both the pre-, or ‘green’, and post-sintered states. The results for *ε_r_* are also plotted in Fig. 3 to better visualize the differences between the polymer-only resins and the silica-based ceramic resins in their unperturbed state, i.e. as manufactured by Formlabs.

**Table 1:** The dielectric permittivity and loss tangent of unloaded Formlabs SLA resins in the W-band at room temperature.

Formlabs Resin	$\varepsilon_{r}$	tan δ
Clear	2.80 ±0.05	0.022 ±0.0020
Black	2.76 ±0.04	0.024 ±0.0012
Gray	2.83 ±0.01	0.022 ±0.0010
White	2.83 ±0.04	0.021 ±0.0010
Draft	2.85 ±0.06	0.019 ±0.0005
Model	2.82 ±0.01	0.023 ±0.0001
High Temperature	2.76 ±0.02	0.020 ±0.0006
Green Ceramic	3.38 ±0.09	0.039 ±0.0013
Post-sintered Ceramic	3.74 ±0.19	0.030 ±0.0027

The results are in excellent agreement with values found in the literature for SLA photoresins at frequencies both above [6,18,[20], [21], [22], [23]] and below [19,24] the W-band. Furthermore, the dielectric permittivity of the pre- and post-sintered ceramic samples is 3.38 ± 0.09 and 3.74 ± 0.19, respectively, both exhibiting *ε_r_* values greater than those from the polymer-only resins due to the large vol % of silica filler material within the photoresin mixture. In fact, the value for the post-sintered ceramic is close to the dielectric permittivity of pure silica (∼3.9) [25], highlighting the likely large degree of densification of the silica network in the sintered ceramic coupon. Despite the utility of these results, the data indicate that all the polymers studied in this work are too lossy for most high frequency applications. Novel resins with exceptionally low loss characteristics must be developed if the high precision capabilities of SLA are to be leveraged by the RF/MW/THz design communities. Even so, these data serve as a reliable reference for the rest of the scientific community.

### 3.2 The dielectric analysis of BST PMCs

**Fig. 4:**
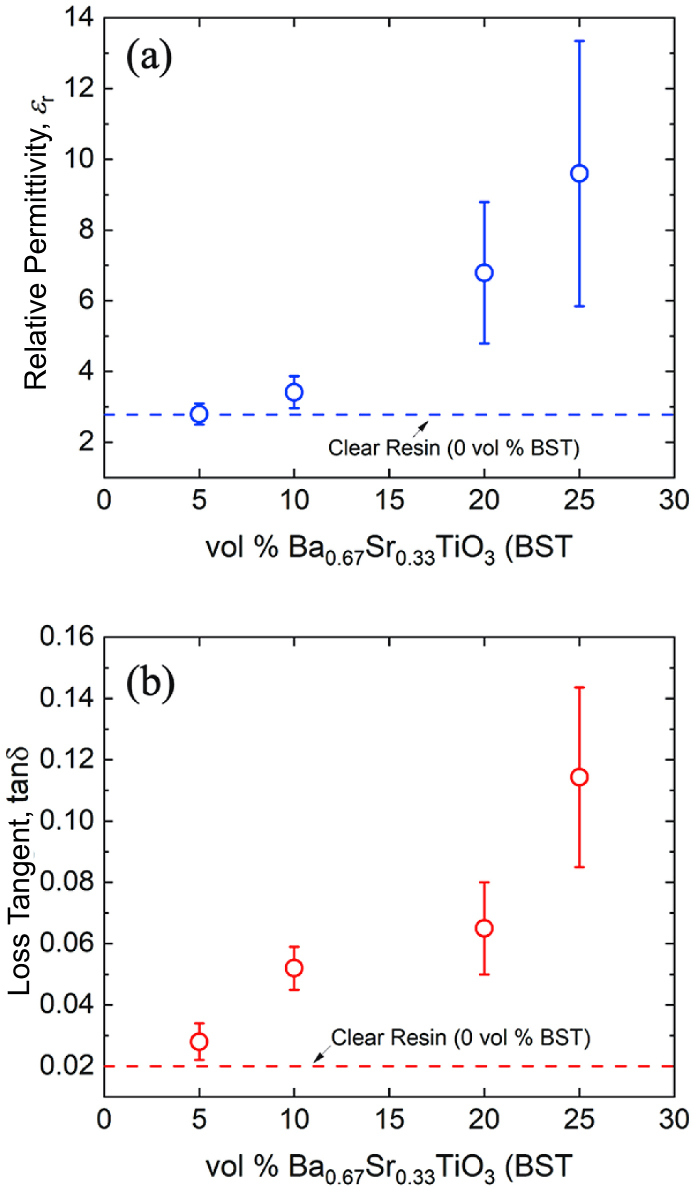
The (a) dielectric permittivity, *ε*_*r*_, and (b) loss tangent, tanδ, of BST-loaded PMC samples as a function of the vol % of BST filler.

Fig. 4 plots (a) *ε_r_* and (b) tanδ for the BST-loaded PMC samples at various volumetric loading concentrations (vol %) of BST, ranging from 5 to 25%. In general, Fig. 4 shows that *ε_r_* increases with increasing BST loading concentration, with the onset of a marked increase in the relative permittivity occurring at volumetric concentrations somewhere between 5% and 10%. At vol % levels greater than 10%, *ε_r_* begins to increase significantly and reaches an average value of ∼9.6 at 25 vol % BST.

As shown in Fig. 4(b), tanδ of the PMCs also increases with increasing vol % BST. In fact, the increase in tanδ is much more abrupt than the increase that is observed in *ε_r_*. For example, tanδ of the PMC increases by ∼150% when BST inclusion is increased from 0 to 10 vol %, whereas the increase in *ε_r_* at the same vol % BST is marginal. Even without the inclusion of BST particles, the photoresins from Formlabs have a tanδ on the order of 10^−2^, and most RF applications require tanδ of underlying component materials to be on the order of 10^−3^ or smaller. Because the loss of these SLA resins is roughly an order of magnitude too large for ideal performance of RF components, research should be devoted to lowering the loss of the underlying polymer matrix before focusing on the relative increase in tanδ with BST particle inclusion. For the purposes of this study, which is concerned primarily with tailoring *ε_r_*, these loss characteristics are important to note but not of central concern.

### 3.3 The Lichtenecker mixture model

The Lichtenecker mixture (LM) model was used in this work to gain insight into the trends that were observed in the *ε_r_* versus vol % BST data displayed in Fig. 4. The LM model, which has been used extensively to study composites [16,24,[26], [27], [28]], contains a logarithmic expression that relates the *ε_r_* values of the matrix and filler materials of a two-part composite mixture; however, it is important to note that when the LM model and other mixture models are used in the literature, the dielectric properties of the nano/micro-particle filler are often assumed to be equal to the bulk property values of the same material [26,27]. This assumption is typically made in lieu of performing an empirical analysis of the filler material and can be quite problematic, as it neglects the effects that size has on many functional properties, including *ε_r_*. These effects are particularly important when characteristic feature sizes/geometries are on the order of 1 μm or smaller. As a result, to properly apply a mixture model to study nano/micro-particle composite mixtures, it is essential to accurately characterize the dielectric properties of the particular filler in question - as a particle, instead of as a bulk material. Unfortunately, dielectric measurements of powders are often more difficult to perform than are similar measurements of bulk materials due to inherent sample preparation differences. For example, when testing a powder material, a volume must be packed with the powder and a density of each sample must be computed, which introduces a degree of uncertainty into the measurement that is not otherwise present when testing bulk materials.

**Fig. 5:**
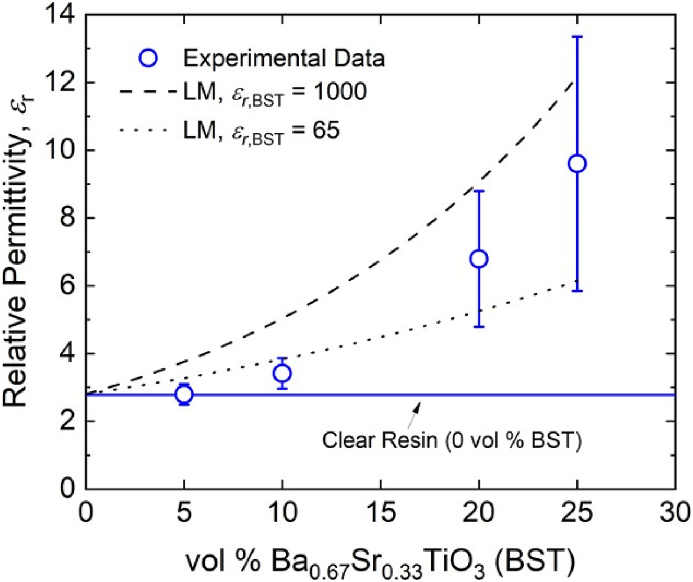
The effect of the assumed ceramic filler properties on the well-known Lichtenecker mixture model.

As an alternative to this procedure, here we use the LM model to gather insight into the actual *ε_r_* of the BST filler used in this work. To enhance the discussion of both the model and the reliability of the data gathered herein, multiple potential values of *ε_r_* were assumed for the BST filler. Fig. 5 plots the LM model assuming two separate values for *ε_r_* of the BST filler (*ε_r_*^*,*^BST = 65 and *ε_r_*^*,*^BST = 1000) along with the experimental data from Fig. 4(a) for comparison. Our particular composition of BST (Ba0.67Sr0.33TiO3) exhibits a Curie temperature slightly above room temperature in bulk form, leading to extremely large values of *ε_r_* on the order of 103–104 [29]. However, the *ε_r_* of the sub-micron (∼200 nm) diameter BST powder used in this work will likely be far lower due to size effects [30,31], bringing the true *ε_r_*^*,*^BST somewhere between the values that were assumed for the cases plotted in Fig. 5. The curves for the LM model highlight the fact that the overall dielectric permittivity of the mixture can vary greatly depending on the assumed properties of the filler.

As shown in Fig. 5, the model overestimates all experimentally determined values of PMC permittivity when *ε_r_*^*,*^BST = 1000, though the shape of the data appears to be well-captured. On the other hand, if *ε_r_*^*,*^BST = 65, the model both over and underestimates the experimental values at low and high loading concentrations, respectively; however, it does appear to provide a reasonable estimate of a low-bound for the expected PMC permittivity at loading concentrations ranging from 5–10 vol % BST. In general, the LM model appears to capture the overall shape of the experimental data, suggesting that accurate prediction of the PMC *ε_r_* versus vol % BST would likely be possible if a precise measurement or estimate of *ε_r_*^*,*^BST was known for this particular nanoparticle paraelectric filler.

### 3.4 Enhancing the printability of the BST PMC

Although the PMC samples demonstrate considerable dielectric functionalization, the BST nanoparticle filler significantly reduces the 3D printability of the resin. This phenomenon has been investigated by other researchers in this field [9]. Numerous studies have shown that ceramic fillers negatively impact the 3D printability of polymer matrices for several reasons. For example, the ceramic filler may reduce the laser curing depth of a 3D printer through the incidental diffraction and absorption of the UV-curing laser light due to a refractive index mismatch between the filler and matrix materials [32].

In the context of this work, the refractive index mismatch between the BST nanoparticles and the photoresin matrix must be considered. Despite efforts to maintain the 3D printability of the BST PMCs, the Form2 SLA 3D printer could not fabricate testable samples from any of the composite mixtures. This particular 3D printer emits a UV laser with a wavelength of approximately 405 nm. At this particular wavelength, the refractive index of the BST nanoparticles and the photoresin matrix used in this work are approximately 2.37 and 1.50, respectively [33,34]. This significant mismatch likely reduced the laser penetration depth of the SLA printer and the overall 3D printability of the mixtures.

In addition to diffraction, the absorption characteristics of the ceramic filler may impact the 3D printability of a PMC. Any fraction of laser curing light absorbed by the filler will reduce the overall printability of the composite. BST has a bandgap of ∼3.4 eV, which is relatively close to the energies of the photons found in the Form2 SLA laser used in this study. Therefore, it is likely that the BST filler used in this work absorbed some degree of the 405 nm light emitted by the Form2 SLA printer, thereby further reducing the laser penetration depth of the UV light source.

Based on these considerations, the printability of the BST PMC could be improved either by including a third material that reduces the refractive index mismatch between the filler and photopolymer or by increasing the overall photosensitivity of the polymer matrix to overcome any incidental absorption by the filler.

Various selection criteria related to the dimensional properties of the ceramic fillers and the chemical compositions of the polymer matrices could also enhance the 3D printability of a PMC. In particular, the ceramic particle size, shape, and material [9] as well as the relative concentration of dispersant and photoinitiator in the polymer matrix [17] could be optimized to improve PMC printability. To this end, the particle size of the filler must be increased to at least the micron-scale and the statistical distribution of particle shape within the powder must be narrowed. If these solutions are optimized, the printability of PMC photoresins containing BST or many other fillers could be enhanced, leading to SLA fabrication of high-performance RF/MW/THz structures. The validation of the enhanced 3D printability due to these considerations is left to future work.

## 4 Conclusions

This work demonstrates the dielectric functionalization of a polymer matrix through the inclusion of BST nanoparticles, where both the dielectric permittivity and dielectric loss of the PMC material increased with increasing BST loading concentration. In addition, dielectric measurements of several commercial SLA photoresins from Formlabs were experimentally gathered and subsequently presented in the text. This work has provided permittivity and loss values in the W-band for use by RF/MW/THz component designers looking to leverage the SLA manufacturing method in their work.

The Form2 SLA 3D printer used in this work was unable to print testable samples with BST PMC composite mixtures. However, the demonstrated dielectric functionalization of the PMC remains an attractive property for the design of unique RF structures. Therefore, an improvement in printability is desired. Building off the suggestions from related work, numerous solutions are proposed to enhance the 3D printability of BST PMCs. For example, to improve the laser curing depth of the Form2 SLA printer, the BST particle size, as well as the dispersant and photoinitiator concentrations, must be increased. Further, pre-processing techniques must be employed to limit the variation in size and shape of the BST particles. In addition to these solutions, a third material could be included in the mixture to reduce the refractive index mismatch between the BST nanoparticles and the polymer matrix. Based on the relevant literature, these solutions would improve the printability of the BST PMC and make it a viable material for the fabrication of RF structures.

## Author contribution statement

Michael Kenneth Forstmeier, M.S.: Conceived and designed the experiments; Performed the experiments; Analyzed and interpreted the data; Wrote the paper.

Mengxue Yuan, Ph.D; Steve Perini; Michael Lanagan, Ph.D: Contributed reagents, materials, analysis tools or data.

Brian Foley, Ph.D: Conceived and designed the experiments; Contributed reagents, materials, analysis tools or data.

## Funding statement

Mr. Michael Kenneth Forstmeier was supported by Center for Dielectrics and Piezoelectrics, North Carolina State University [1841453 & 1841466].

## Data availability statement

Data will be made available on request.

## Declaration of interest’s statement

The authors declare no competing interests.
